# Analysis of an explanted dual mobility cup after 21 years, parabolic wear pattern: A case report

**DOI:** 10.1051/sicotj/2022052

**Published:** 2023-01-17

**Authors:** Michel-Henri Fessy, Arthur Fessy, Anthony Viste

**Affiliations:** 1 Hospices Civils de Lyon, Hôpital Lyon Sud, Chirurgie Orthopédique et Traumatologique 165 Chemin du Grand Revoyet 69495 Pierre Bénite Cedex France; 2 Univ de Lyon, Université Claude Bernard Lyon 1, Univ Gustave Eiffel, IFSTTAR, LBMC UMRT_9406 Lyon France; 3 INSA 20 Av des Buttes de Coesmes Rennes France

**Keywords:** Dual mobility, Wear, Total hip arthroplasty

## Abstract

*Case*: A dual mobility cup was implanted in 1983 in a 43-year woman. After 31 years of normal function, we analysed the explanted materials with modern techniques. *Conclusion*: Volumetric wears of the small and large articulations of the dual-mobility construct were similar. For the first time, we demonstrated that the dual-mobility liner underwent parabolic (not linear) wear during the period of implantation.

## Introduction

In 1983, Mrs E. underwent total hip replacement on her right side at 43 years of age. This procedure was performed by Professor Gilles Bousquet for osteoarthritis due to hip dysplasia. At the time, Bousquet implanted a tripod metal-backed shell (cup size 45 mm, liner CIC size 45/22 mm) (SERF, Decines, France) with two anchoring pegs and one screw. A femoral stem from the same manufacturer was implanted: a 22 mm one-piece stainless steel head and neck having a modular base with a screwed stem (EC 22 neck and MCB base). She had excellent outcomes with this procedure and forgot about her hip until the year 2000, at which point she developed spontaneous pain. Radiographs showed expansile osteolysis in the superior pubic ramus, facing the anchoring peg. The bone scan showed localized uptake in this area, evidence of a fracture secondary to granuloma, while the implants remained firmly in place. Functional treatment in which the patient used a cane for 45 days eliminated the pain, which allowed her to resume unlimited walking without a cane and without limping. Given that the bone scan showed no problems with fixation, the implants were not revised.

**Table 1:** Wear measurements of the liner CIC 45/22 studied.

	CIC 45/22
3D volume	16,307.51
Theoretical maximum volume	19,918.70
Theoretical mean volume	19,733.40
Theoretical minimum volume	19,541.60
Maximum wear	18.13%
Mean wear	17.36%
Minimum wear	16.55%

**Figure 1:**
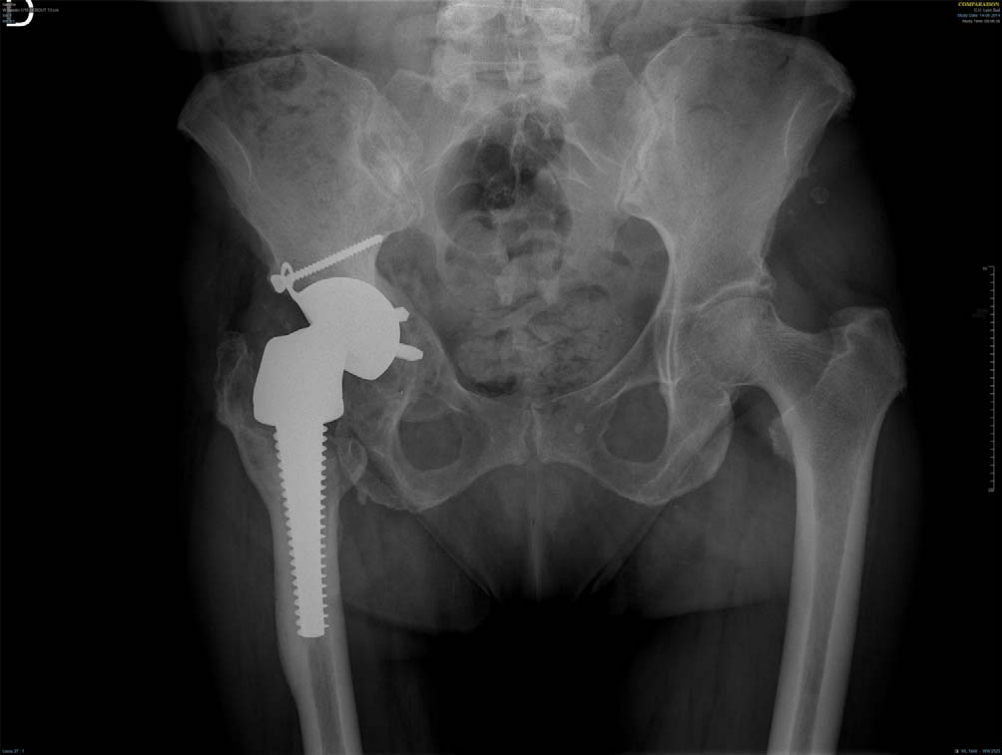
AP view of the pelvis taken in 2014.

In 2014, cup aseptic loosening was diagnosed (Figure 1) leading to unipolar acetabular revision. The dual mobility cup was explanted and analysed in 2020 with modern, highly accurate analysis methods.

## Statement of informed consent

The patient was informed that data concerning the case would be submitted for publication and agreed.

## Case report

The explant was examined macroscopically before and after separating the head and polyethylene liner. The head was examined with a 3D coordinate measuring machine. The separated parts were then digitized according to a protocol previously described by Boyer et al. [1].

The explant was analysed in three dimensions using a structured-light 3D scanner (Zfx Evolution, Zfx GmbH, Dachau, Germany) with an accuracy of <9 μm. During acquisition, the explant was covered with a layer of white powder 3–5 μm thick to prevent it from reflecting the green light. Using various positions, the two cameras (1296 × 964 resolution, CCD chips) captured all the points on the surface of the explant needed for digitisation. The explant was analysed in two different positions. The two resulting files were merged using Meslab^®^ software (ISTI – CNR, Pisa, Italy) using a minimum of four pairs of identical points in each file.

To be able to analyse this file in Pro-Engineer^®^ (PTC Inc, Boston, MA, USA), reconstruction by the Poisson algorithm was done to fill in the holes and produce an STL file of the entire explant. For the last step – solidification – the STL file was converted into a solid shape by the Pro-Engineer^®^ software. This provides a model of the explant with an accuracy of about 10 μ. After digitisation, the explant was analysed with CREO 3D CAD software (version 2.0, PTC).

## Macroscopic analysis (Figure 2)

Impingement lesions were found between the prosthetic base and the metal-backed shell causing deformation of the shell with burrs. However, this did not prevent the PE liner from moving freely inside the metal shell. A circular deterioration pattern due to neck impingement was found around the orifice where the head is introduced into the PE liner. There was no intraprostatic dislocation. On the outer surface, there was a dark band at the level of the anchoring pegs. Residual metal debris was found on the load-bearing areas of the PE liner. A linear notch was visible parallel to the free edge of the PE liner in the load-bearing area.

**Figure 2:**
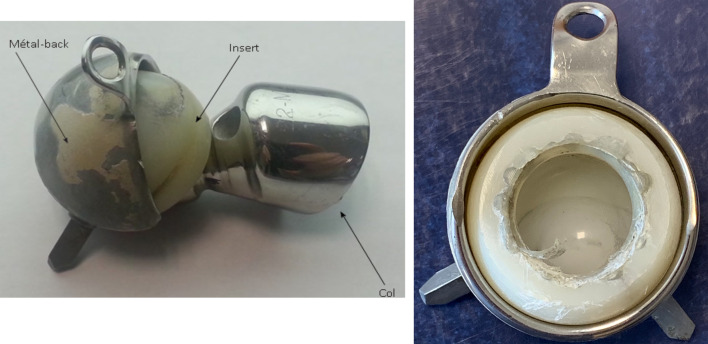
Photograph of the explant analysed.

## Analysis of digitized components in CREO

### PE liner

The explant was compared with the manufacturer’s tolerance ranges for this type of liner. Based on our calculations, the liner underwent 16% to 18% wear.

**Figure 3:**
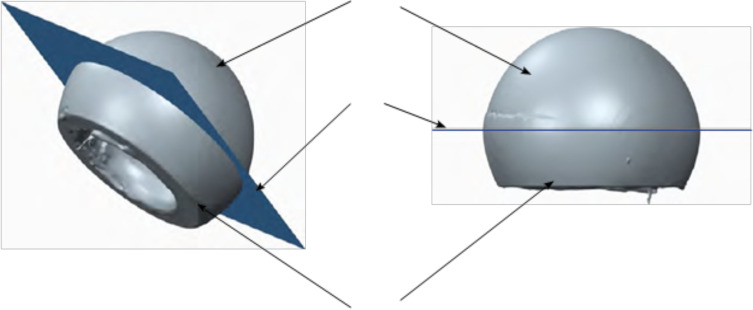
Plans of study.

**Figure 4:**
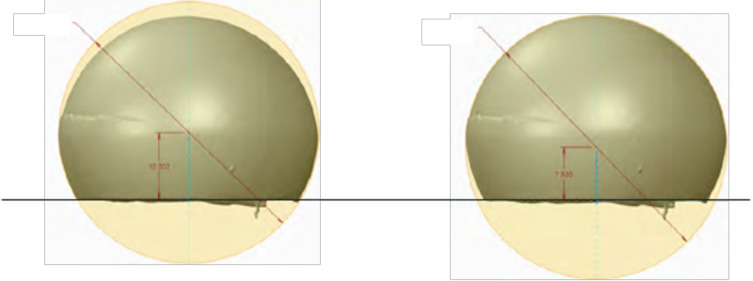
Linear wear of the large articulation was 2.1 mm.

**Figure 5:**
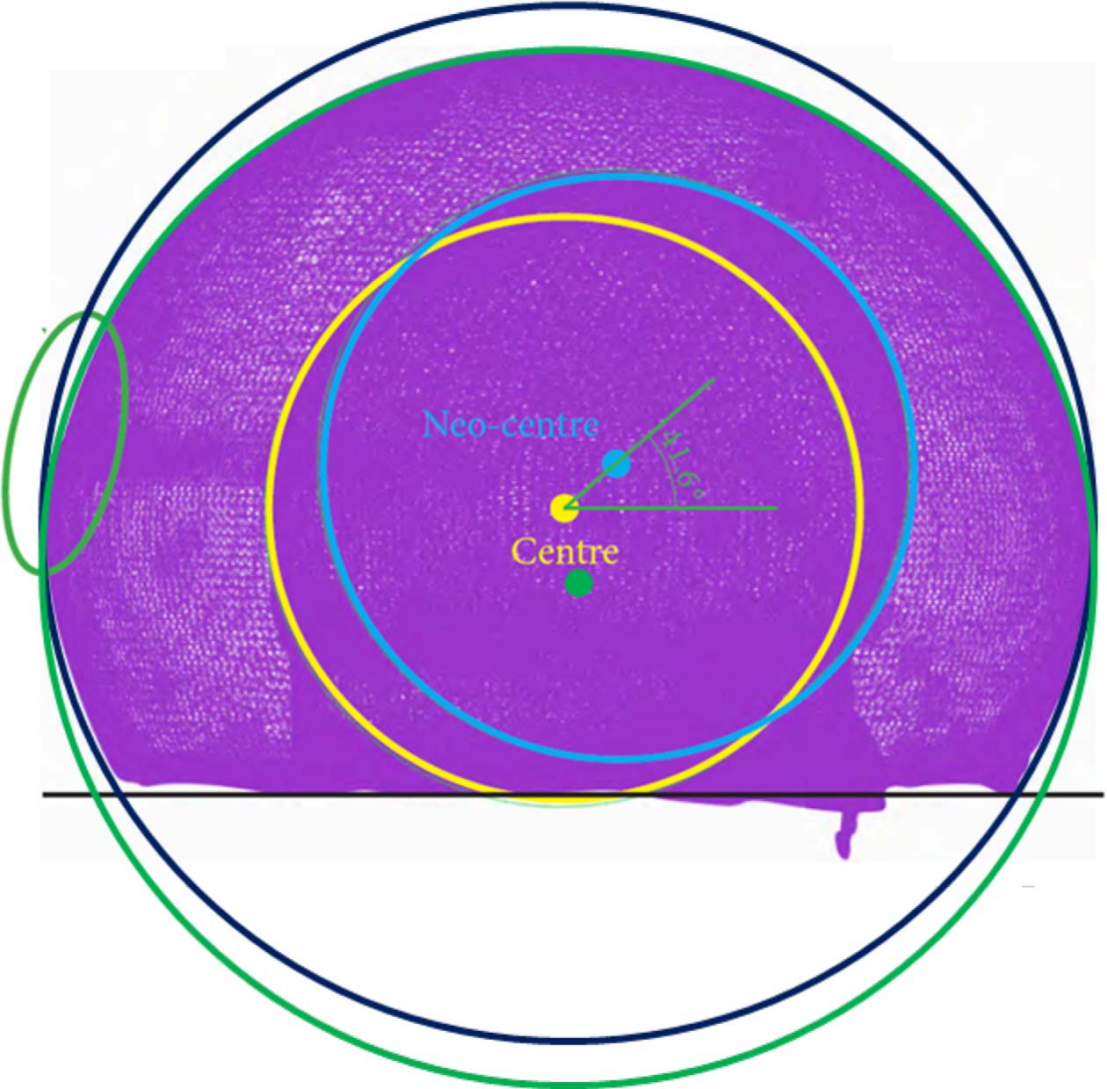
Internal (2.5 mm) and external wear (2.1 mm) of the polyethylene liner. Liner centre (in green), native head centre of the construct at the implantation (in yellow), and new head centre of the explant after wear (in blue).

Analysis of the outer surface identified two zones on the liner, separated by a plane parallel to the flat portion of the liner and passing through its centre (Figures 3 and 4). The analysis indicated that the head had migrated a considerable distance towards a new centre, offset by 2.6 mm. The offset angle was 41.6° in the plane closest to the reference plane, which is the liner’s free plane (Figure 5).

### Metal-backed cup

**Figure 6:**
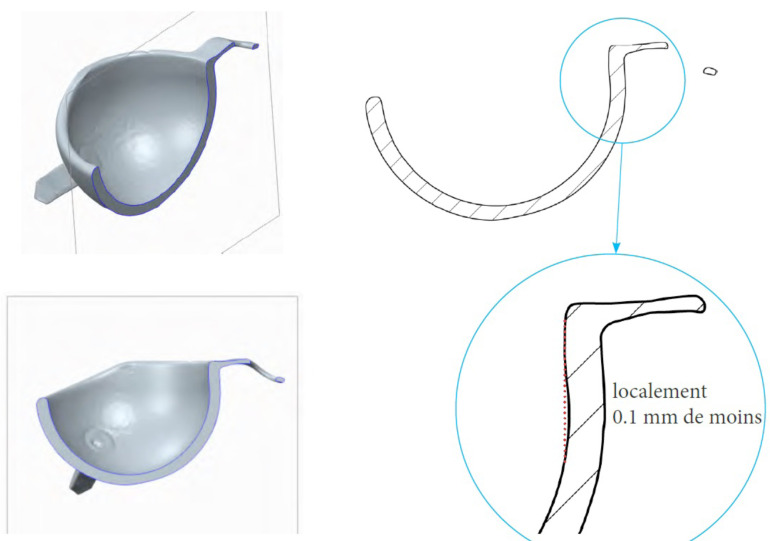
Wear of the metal-backed shell.

**Figure 7:**
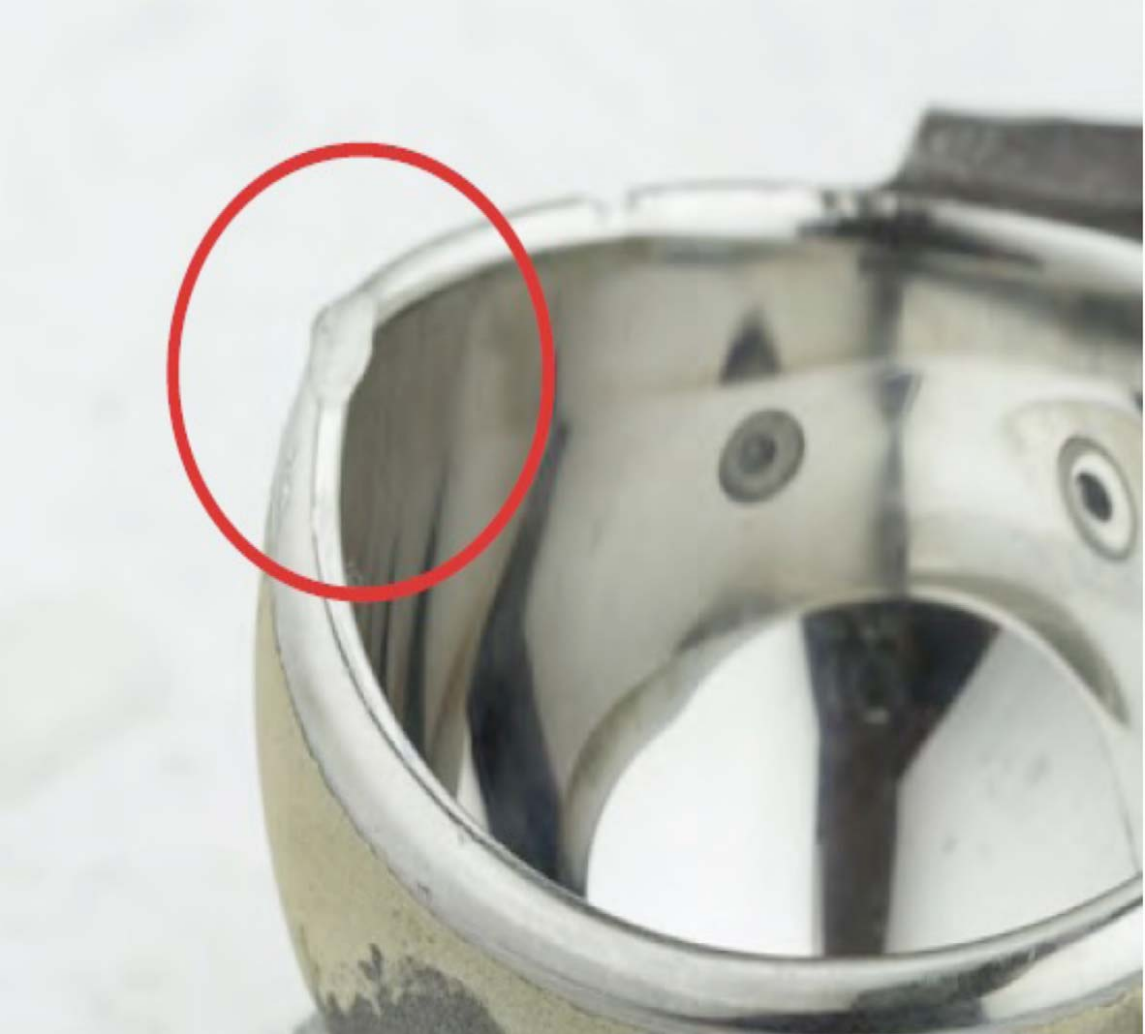
Impingement of the neck on shell.

PE liner caused deformation of the shell at the equator. This deformation was in the area when the stresses were the largest (Figure 6). An area of friction was found at the inferior left peg and may explain the stripe deformation observed on the lower portion of the liner (Figure 5). The periphery of the shell was deformed, with two flat spots caused by impacts between the neck and shell. The largest deformation was on its posterior side. This deformation created a burr inside the shell, which likely contributed to liner wear in this area (Figure 7).

### Head, neck, metaphysis

**Figure 8:**
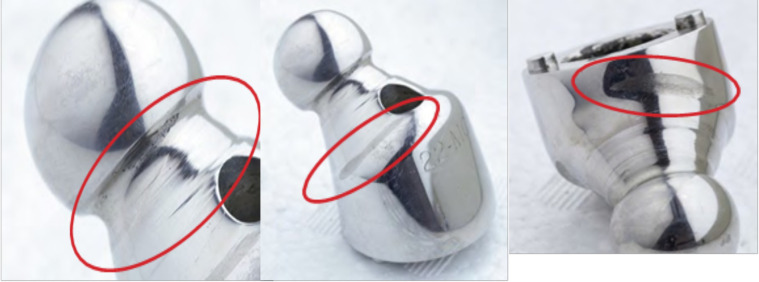
Deformations found on neck.

The head was no longer spherical. The mean diameter of the sphere was 21.6 mm (0.4 mm wear). Three areas on the neck were deformed (Figure 8). The first was below the head, near the impaction hole, where the neck rubbed against the liner as the head rotated within the liner. The second area was located on the superior and posterior edges of the neck. These marks correspond to impingement between the neck and shell during extension. The repeated impact created a burr on the shell. The last area was located under the head on the anterior side of the neck. These marks correspond to impingement between the neck and shell during hip flexion (seated position) and generated a flat spot on the metal shell.

## Discussion

Dual mobility cups are an extremely popular medical device given their high level of effectiveness [2–5]. This novel medical device has proven long-term fixation and survival [2, 4]. While this device is widely used now, we do not know much about how it functions. To our knowledge, it is the first description of the parabolic wear pattern of a dual-mobility construct. For this reason, analysing explants [1, 6–9] that have functioned normally inside the body for several years is essential for perfecting our knowledge, even if we cannot draw statistical conclusions from it.

Based on our analysis, three contact points triggered wear and deformation: head/liner, liner/cup, and neck/cup.

Analysis of dual mobility systems is often limited to looking at the wear of the PE liner [6]. In our explanted device, the liner played a minimal role in the deformation and wear of the metal shell, along with the wear of the head and neck.

### Neck-cup wear

The periphery of the cup was damaged due to impingement with the neck. This can be explained by the hemispheric cup’s overhanging design, but also by the poor relationship between the head and neck of the femoral stem, since the base is relatively bulky in this model (16 mm diameter) resulting in a head-to-neck ratio of 1.37 given the 22-mm head. This impingement undoubtedly contributed to the implant shifting, which initiated loosening.

### Liner-cup wear

Despite the soft nature of the PE liner, we found the metal shell to have 0 to 1 mm of wear in the load-bearing zone, leading to metal ion release, which may contribute to polyethylene tattooing and increased wear due to abrasion from a third-body effect. The PE liner was damaged by a slight protrusion of the peg, causing a circular notch (Figure 5).

### PE liner wear

We observed wear of the PE liner at the small articulation between the femoral head and PE liner, and the large articulation between the PE liner and cup.

In our case, the linear wear of the small articulation was 2.5 mm (Figure 5) while the wear of the large articulation was 2.1 mm (Figures 4 and 5). In this case, linear wear totalled 4.6 mm over 21 years (0.22 mm per year). Conversely, like in the study by Adam et al. [6], linear wear was similar between the small and large articulations.

### Head-liner (small articulation) wear

**Figure 9:**
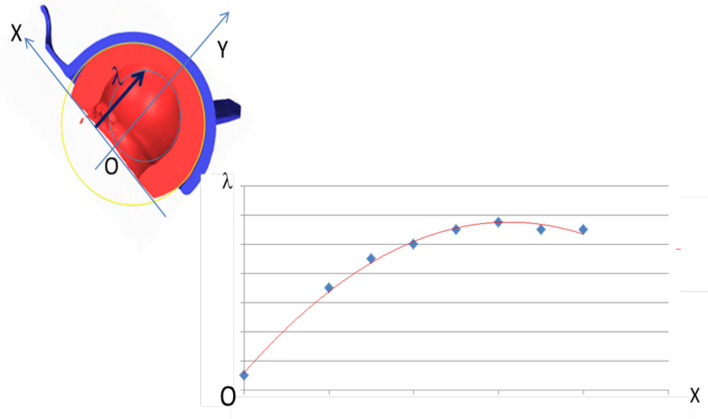
Wear is parabolic, not linear.

**Figure 10:**
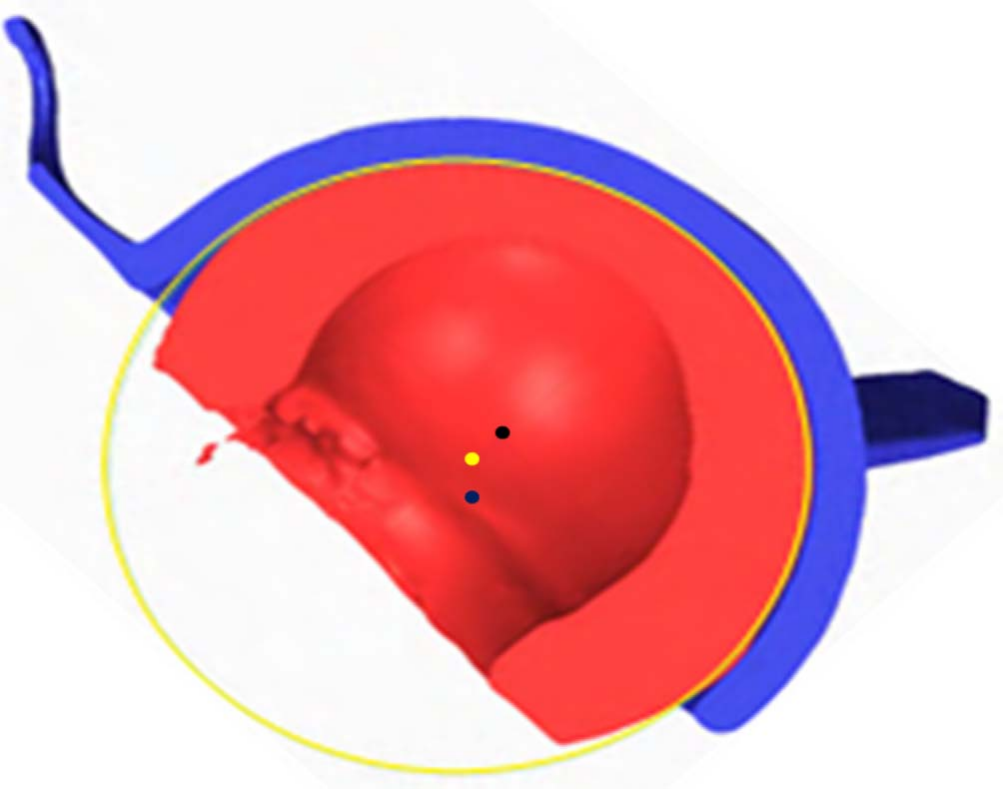
Shifting of the centres of rotation (yellow dot: cup’s centre of rotation, black dot: head’s centre of rotation, blue dot: liner’s centre of rotation).

The wear of the small articulation was not linear, contrary to the findings with standard fixed-cup designs. A more precise analysis of the small articulation showed that its wear followed a parabolic curve, which started to erode the retaining zone from the inside (Figures 9 and 10).

Thus, it appears that wear of the retaining zone was triggered by impingement with the prosthetic neck, but also by progressive subluxation of the PE liner during parabolic wear of this small articulation. This is an original and brand-new finding.

### Centres of rotation

At the time of implantation, the centre of rotation of the cup, head and PE liner are all superimposed (Figure 5, yellow point). However, these three centres shifted over time: the centre of the cup did not move; the centre of the head followed a parabolic path if the PE liner is considered as a frame of reference (Figure 5, blue point); the centre of rotation of the PE liner shifted downward along the trajectory of the resultant of the loads placed on the hip (Figure 5, green point).

Figure 10 shows the configuration in which the liner and shell should have been when they were explanted, versus the configuration in which we found them.

## Conclusion

Volumetric wear of the small and large articulations was similar. Nevertheless, the dual mobility cup that we analysed underwent a period of parabolic wear over the 31 years it was implanted.

## Author contrubutions

MHF: draft, writing, editing. AF: draft, writing. AV: drat, writing, editing.
